# Soil moisture and soil organic carbon coupled effects in apple orchards on the Loess Plateau, China

**DOI:** 10.1038/s41598-024-63039-2

**Published:** 2024-05-29

**Authors:** Lei Han, Guowei Nan, Xinyu He, Jinghui Wang, Jirong Zhao, Xiangqian Zhang

**Affiliations:** 1 School of Life Sciences, Yan’an University, Yan’an, 716000 China; 2 Engineering Research Center of Microbial Resources Development and Green Recycling, University of Shaanxi Province, Yan’an, 716000 Shaanxi China

**Keywords:** Soil moisture, Soil organic carbon, Coupled effect, Northern Shaanxi, Apple orchards, Forest ecology, Carbon cycle, Environmental impact

## Abstract

A large number of economic forests, especially apple orchards (AOs) in the Loess Plateau region of China, have been planted to develop the local economy and increase the income of farmers.

The two main constraints preventing AOs on the Loess Plateau from developing sustainably and producing a high and steady yield are soil moisture content (SMC) and soil organic carbon (SOC). Nevertheless, little is currently known about the contributions of roots to these changes in the soil profile and the temporal modes of the SMC-SOC coupled effects. In our research, we analyzed the dynamic changes in SMC and SOC in AOs of various years in northern Shaanxi Province, as well as the coupled relationship between the two, and attempted to describe the function of roots in these changes. Research have shown: (1) As the age of the AOs increased, the SMC continued to decline throughout the 0–500 cm profile, especially at depths of 100–500 cm. SMC depletion mainly occurred in AOs aged 20 years (30.02%/year) and 30 years (31.18%/year). (2) Compared with abandoned land (AL), all the AOs except for the 6-year-old AO showed a carbon sequestration effect, and the carbon sequestration effect increased with age. The carbon sequestration rate of the 12-year-old AO was the highest and then decreased with age. Both surface and deeper soils showed better carbon sequestration, with a large amount of SOC being sequestered in deeper soil layers (> 100 cm). (3) The coupled effects of SMC and SOC varied with age and depth. The SMC in the deeper layers was significantly negatively correlated with SOC. Root dry weight density (RDWD) was significantly negatively correlated with SMC and significantly positively correlated with SOC. Path analysis suggested that SMC directly affects SOC at different soil depths, and regulates SOC by affecting RDWD, but these effects are significantly different at different depths. Therefore, we propose that management of AO should focus on the moisture deficit and carbon sequestration capabilities of deeper soils to ensure the sustainability of water use in AOs and the stability of agricultural carbon sequestration on the Loess Plateau.

## Introduction

Shaanxi apples are from one of the largest and finest apple growing areas in the world^1,2^. The northern Shaanxi region has long been a preferred apple production area due to its unique natural conditions. Although apple orchards (AOs) are the most dominant and competitive industry in the region's rural economy, their productivity sustainability is still profoundly limited by water scarcity^3^.

In the water-scarce Loess Plateau region, water shortages constrain apple production and quality^4,5^. The AOs in the region rely largely on rainfall for soil moisture content (SMC) recharge^6,7^. In northern Shaanxi, apple industrialization has progressed rapidly in the last few years, and changes in cropping patterns and the refinement of management practices have greatly improved apple yields. However, apple trees also greatly decrease soil moisture and soil fertility^8,9^. In addition, the drying and warming climate on the Loess Plateau and the commonly occurring soil desiccation in AOs further increase the instability of fluctuations in apple yield with rainfall changes^10^ and cause many agricultural ecological problems (e.g. decreases in fruit tree growth, yield, quality, soil fertility, the fruit setting rate, disease and insect resistance). Soil desiccation in AOs not only reduces the yield and quality of apples and affects the current sustainable production capacity of AOs but also has a significant impact on newly planted orchards after the replacement of old orchards, seriously constraining the health and stability of the apple industry in northern Shaanxi^10,11^. For example, Huang et al.^10^ confirmed that before the emergence of the soil desiccation, the fluctuation of inter-annual rainfall did not have much effect on apple tree yield, but after the emergence of the soil desiccation, the yield of apple trees showed greater fluctuation with the change of inter-annual rainfall. Zhang et al.^11^ reported that with the extension of growing years, the soil moisture in the 0–10 m soil layer of apple orchards gradually decreased, and the distribution depth of the soil dry layer gradually increased, while the yield of apple orchards as a whole showed a fluctuating trend of decline with the increase of apple years. Guo et al.^12^ found that the higher the planting density, the faster the yield growth of the orchard in the initial stage, and later on the yield decline gets larger due to high soil moisture overdepletion. Thus, clarifying the dynamic characteristics of SMC at different ages in AOs in northern Shaanxi is important for sustainable soil moisture management in AOs in the future.

Soil organic carbon (SOC) is essential for forming and maintaining soil structure, nutrient cycling, and biodiversity^13–15^. SOC is the world's largest and most active carbon pool, and small changes in SOC can significantly affect the global carbon balance in both natural and human systems^16^. Agricultural soils can offset one-fourth to one-third of the annual increase in atmospheric CO_2_ estimated at 3.3 Pg C per year^14^. Thus, the carbon sequestration function of agricultural soil is regarded as a valid way to alleviate global warming^15^. In the context of China's current dual carbon strategy, strengthening the research on the carbon sequestration function of agricultural soil will not only help to restore China's degraded soil fertility, but will also help to protect China's developing industries in regard to future greenhouse gas control negotiations. SMC has a strong coupled relationship with SOC^17^, which mainly affects the retention effect of SOC by regulating the input (litter), transformation (soil microorganisms) and output (soil respiration) of SOC^18–20^. In particular, in the water-scarce Loess Plateau region, the soil carbon cycle is more sensitive to reductions in soil moisture^21,22^. The apple tree is a deep-rooted tree species, which induces a regional deep soil moisture deficit that directly or indirectly affects the sequestration effect of SOC to a certain extent^23^, increasing the uncertainty of its soil carbon sequestration function^24,25^. For instance, Li et al.^26^ results showed that the conversion of long-term cropland to AOs did not cause significant changes in soil organic carbon. Yang et al.^27^ showed that AOs have lower SOC sequestration values than cropland. Li et al.^14^ confirmed that water scarcity limited soil organic carbon sequestration in old AOs in loess-covered areas. Therefore, while clarifying the soil moisture deficit characteristics of AOs in Shaanxi Province, we should consider discovering the sequestration effects and relationships between the two, which is meaningful for accurately quantifying the carbon sequestration capacities, stability and potentials of agricultural soils in the region.

The Loess Plateau region has undergone relatively extensive research on soil moisture deficit and soil carbon sequestration, but existing major research has concentrated on the soil moisture-carbon effect of afforestation, and most research on AOs has concentrated on shallow soils (< 1 m). Additionally, there is a lack of soil moisture and carbon studies at the actual depth (below 1 m) where soil moisture deficits occur, as well as explorations from different stand ages and soil depths. We hypothesized that the distribution of SMC and SOC in the 0–500 cm profile changed after the AOs were planted, but the range of changes varied with AO age. Therefore, the aims of our research were (1) to investigate the dynamic characteristics of SMC and SOC in different years of AOs at depths from 0 to 500 cm and (2) to research the coupled relationship between SMC and SOC at different ages and soil depths. This research has some practical value for the management of AOs in the Loess Plateau region and similar regions of the world.

## Materials and methods

### Research area overview

**Figure 1:**
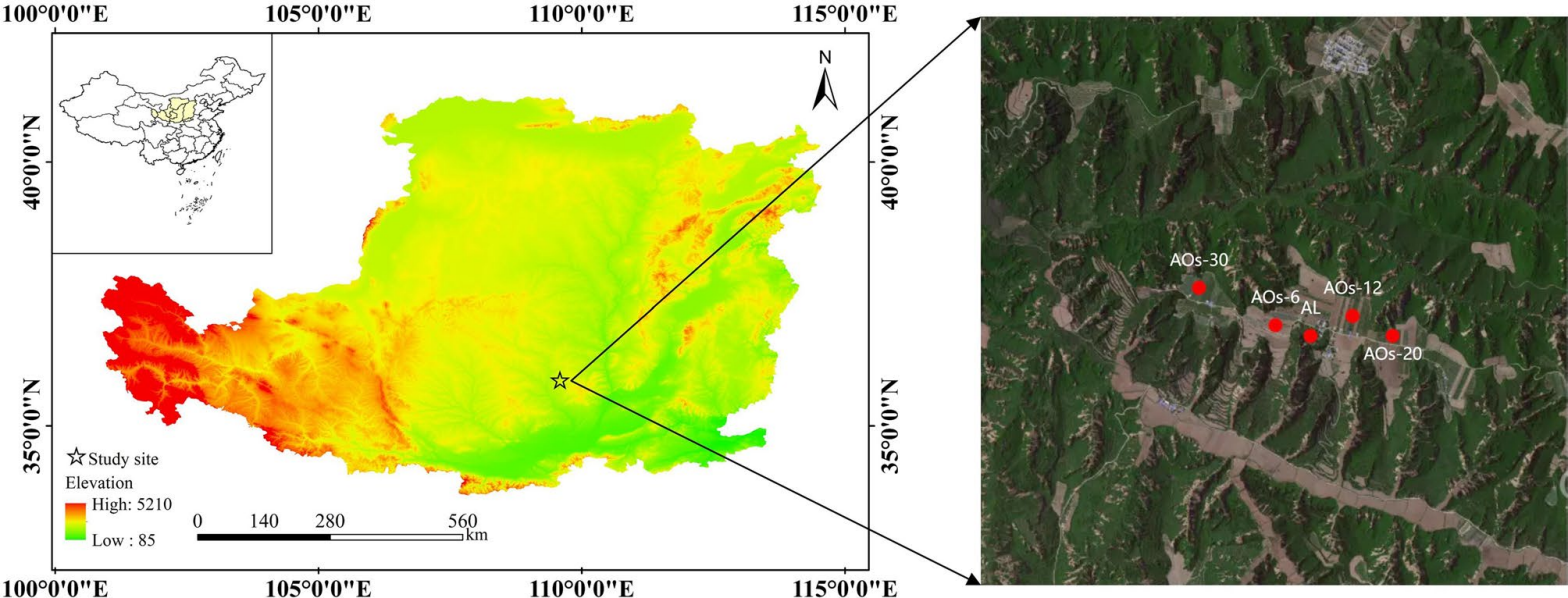
The location of the study site.The red point within the right figure represents the specific sampling locations (Note: The map was generated using ArcMap 10.6 software, URL: https://www.arcgis.com/index.html; Tianditu, https://map.tianditu.gov.cn/).

The research area was located in Luochuan County (Fig. 1), Yan'an City, Shaanxi Province, China (35° 30′ 32′′ N, 109° 26′ 15′′ E), which is a typical apple cultivation base on the south-central part of the Loess Plateau, and the altitude is 1100 m. The average sunshine hours in the area are 2418.8 h, and the frost-free period is up to 180 days. The average annual temperature is 9.2 °C, the average rainfall is 622 mm, and the total annual radiation reaches 55.41 k J cm^-2^. In addition, the study area is characterised by a typical loess landform. The soil types in the apple orchard mainly consist of black clay and loess, and these soil profiles are homogeneous and deep. The main orchard species is arborised red Fuji (*Malus domestica* (Borkh.) CV. Red Fuji).

### Experimental design and sampling

Our research mainly used spatial distribution instead of a time series approach, which is commonly used in ecology, to compare and analyze changes in the dynamics of SMC and SOC in AOs of different tree ages. Ages of AOs were determined through visits to local farmers who were the actual owners of these AOs. AOs aged 6, 12, 20 and 30 years (hereafter referred to as AOs-6, AOs-12, AOs-20, and AOs-30, respectively) and abandoned land(AL) aged 6 years were selected as control plots for soil sampling. To more precisely compare soil carbon-moisture effects at these sites, all sites had similar stand conditions (such as elevation, slope, and aspect). In August 2022, we collected soil samples (*n* = 1500) from the 0–500 cm profile at sites of different ages. There were three replicates for each age, and five randomly selected sample points were mixed into one soil sample per plot. All AOs were planted with Fuji trees at a density of 3 m × 4 m. We collected soil samples at 20 cm intervals using a soil auger, placed them in aluminium boxes, and dried them at 105 °C for 12 h to determine their mass SMC (%). Three sampling points were established within 0.5 m to 1.0 m of the tree trunk using a 10 cm internal diameter root auger, with each layer of cores collected at the same interval depth as the SMC. All soil samples were naturally air-dried for SOC content analysis. All root segments in the cores were removed, returned to the laboratory, soaked and rinsed in deionized water to remove soil and contaminants, and undamaged live roots with a diameter of < 2 mm were collected, weighed after drying at 60 °C and then converted to fine root biomass per unit area. The root dry weight density (RDWD, g cm^–3^) was obtained by dividing the root dry weight within the soil taken by the volume of the soil collected.

### Data calculation and analysis

Soil moisture deficit effect (SMDE) is used to assess the effect of different age AOs on SMC.

1 $$ SMDE_{j,k} = \frac{{SMC_{j,k} - SMC_{0,k} }}{{SMC_{0,k} }}, $$

2 $$ SMD{ - }\theta_{j,k} = \frac{{SMDE_{j,k} }}{{\text{T}}}, $$

where SMDE_*j,k*_ is the soil moisture deficit effect in AOs of age j in layer k, SMC_*j,k*_ is the soil moisture content (%) in AOs of age j in layer k and SMC_*0,k*_ is the soil moisture content (%) in layer k of the control AL, SMD-*θ*_*j,k*_ is the rate of soil moisture depletion and T is the age of the AOs.

Soil organic carbon sequestration effect (SOCSE) is used to assess the carbon sequestration capacity of AOs of different ages.

3 $$ SOCSE_{j,k} = \frac{{SOCS_{j,k} - SOCS_{0,k} }}{{SOCS_{0,k} }}, $$

4 $$ SOCS{ - }\theta_{j,k} = \frac{{SOCSE_{j,k} }}{{\text{T}}}, $$

where SOCSE_*j,k*_ is the soil carbon sequestration effect in AOs of age j in layer k, SOCS_*j,k*_ is the SOC content (g/kg) in AOs of age j in layer k, and SOCS_*0,k*_ is the SOC content (g/kg) in layer k of the control AL, SOCS-*θ*_*j,k*_ is the rate of SOC sequestration and T is the age of the AOs.

### Statistical analysis

Descriptive statistics were used to analyze the distribution and changes of SMC, SOC and its roots under different years of AOs, and one-way ANOVA, multiple comparisons, and regression analysis were used to research the difference in SMC and SOC change of AOs in different ages (*p* < 0.05). The IBM SPSS 25.0 (SPSS Inc., Chicago, IL, USA) was used to complete all types of analyses.

## Results

### Characterization of root parameters with stand age

**Figure 2:**
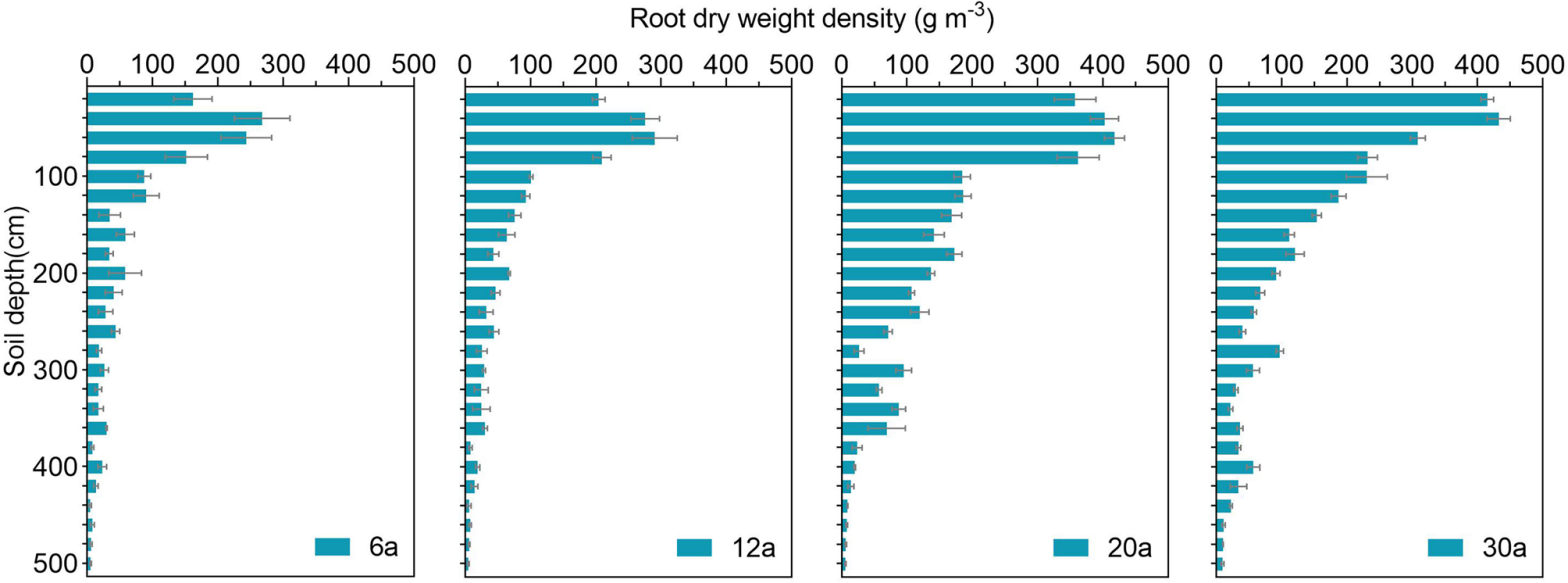
Vertical distribution of root dry weight density (RDWD) in the 0–500 cm soil profile in AOs of different stand ages. Error bars represent the standard error of the mean (n = 3).

The RDWD in AOs changed with age and soil depth (*p* < 0.01), with a general trend of AOs-20 > AOs-30 > AOs-12 > AOs-6, and the RDWD decreased with increasing soil depth at different ages (Fig. 2). The RDWDs of the different age groups were mostly distributed from 0 to 200 cm soil depth (77.8%-81.3%), but the occupation rate of the total RDWDs from the 200 to 500 cm soil depth was still between 18.7 and 22.2% (Fig. 2). The AOs-20 and AOs-30 had significantly greater RDWD than did the younger AOs-12 and AOs-6. AOs-30 had greater RDWD at the 0–100 cm depth, which was greater than that of AOs-20 but lower than that of AOs-20 at the 100–200 cm depth.

### Profile distribution and deficit effect of SMC with different stand ages

**Figure 3:**
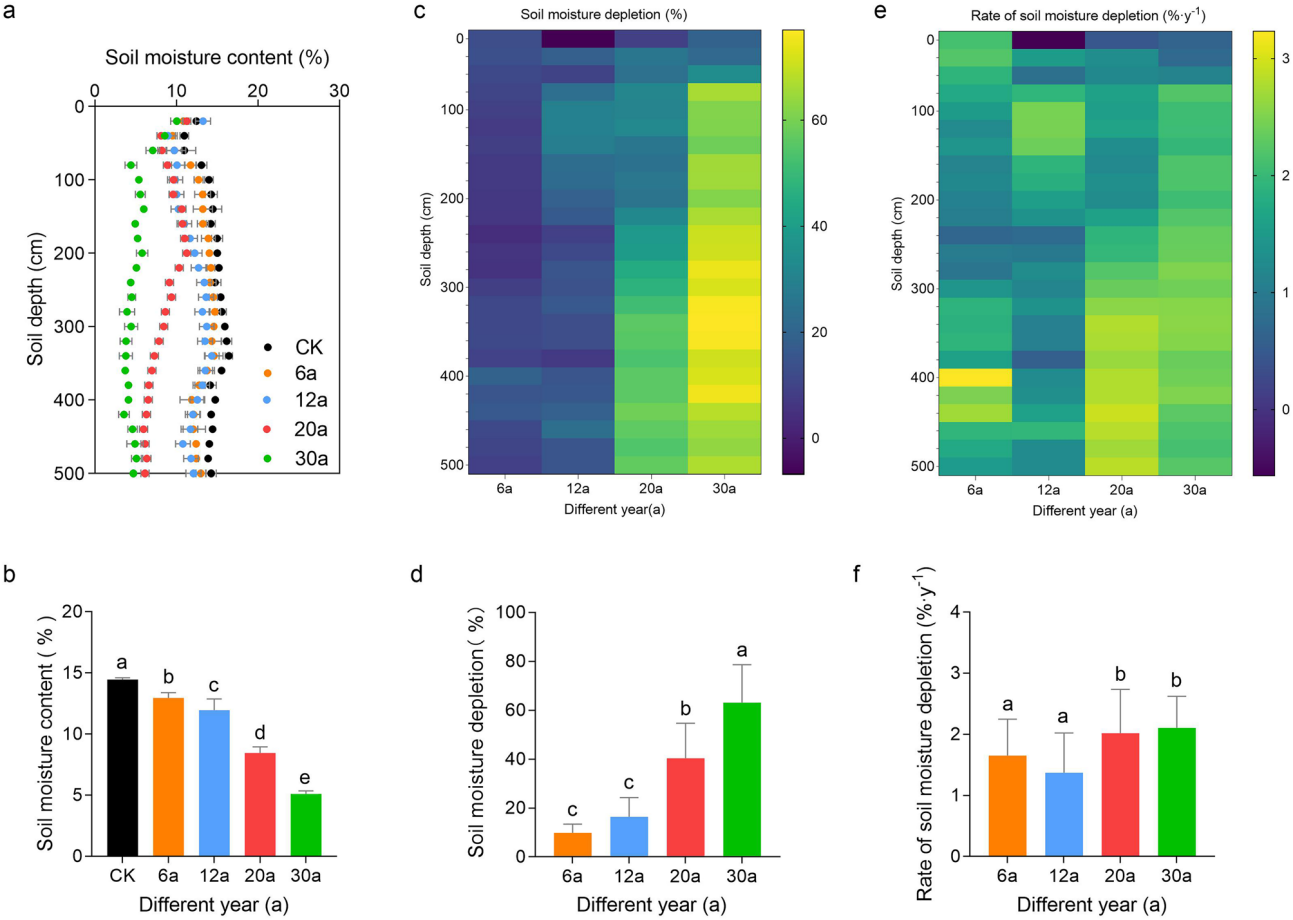
Vertical distribution of the SMC content (**a**,**b**), SMC changes(**c**,**d**) and the rates of the SMC changes (**e**,**f**) in AOs of different stand ages. Error bars represent the standard error of the mean (n = 3).

The distribution of SMC profiles in AOs was influenced by age and soil depth (*p* < 0.01, Table 1). Throughout the 0–500 cm profile, the average SMC values generally decreased with increasing age and were lower than the mean SMC in the AL (*p* < 0.05; Fig. 3a,b).

**Table 1:** Results of the ANOVA on effects of stand age and soil depth on soil organic carbon, soil moisture, and root dry weight density (RDWD).

Characteristic	Age		Depth		Age × depth	
	F-value	p-value	F-value	p-value	F-value	p-value
Soil moisture	1748.0	< 0.0001	14.78	< 0.0001	13.23	< 0.0001
Soil organic carbon	34.41	< 0.0001	5.613	< 0.0001	0.7650	0.9057
Root dry weight density	482.0	< 0.0001	647.8	< 0.0001	22.12	< 0.0001

Figure 3c shows that the soil moisture deficit across the 0–500 cm soil profile of AOs of different ages varied (9.91–63.13%) and increased with age compared with that in the AL. The soil moisture deficit in the AOs-6 was lower. In the AOs-12, soil moisture deficit occurred mainly in the 80–160 cm soil layer, and in the AOs-20 and AOs-30, it was in the 60–500 cm range and increased with depth. Compared to AOs-6, AOs-12 showed an increase in soil moisture deficit, but this increase was not found to be statistically significant (*p* > 0.05; Fig. 3d). In contrast, soil moisture deficit was statistically significant in the remaining AOs at different ages (*p* < 0.05; Fig. 3d).

Regarding the rate of soil moisture deficit, AOs of different ages showed different levels of soil moisture deficit in the 0–500 cm soil depth profile (0.66–5.78%, Fig. 3e), which fluctuated with increasing age (Fig. 3f). Soil moisture deficit rates were lower in AOs-6, greater in AOs-12 at 80 ~ 160 cm, and greater in AOs-20 and AOs-30 at 240–500 cm and 80–180 cm respectively. Overall, the soil moisture deficit rates were greatest in AOs-20, followed by AOs-30, and lowest in AOs-6 and AOs-12 (*p* > 0.05; Fig. 3f).

### Profile distribution and sequestration effect of SOC with different stand ages

**Figure 4:**
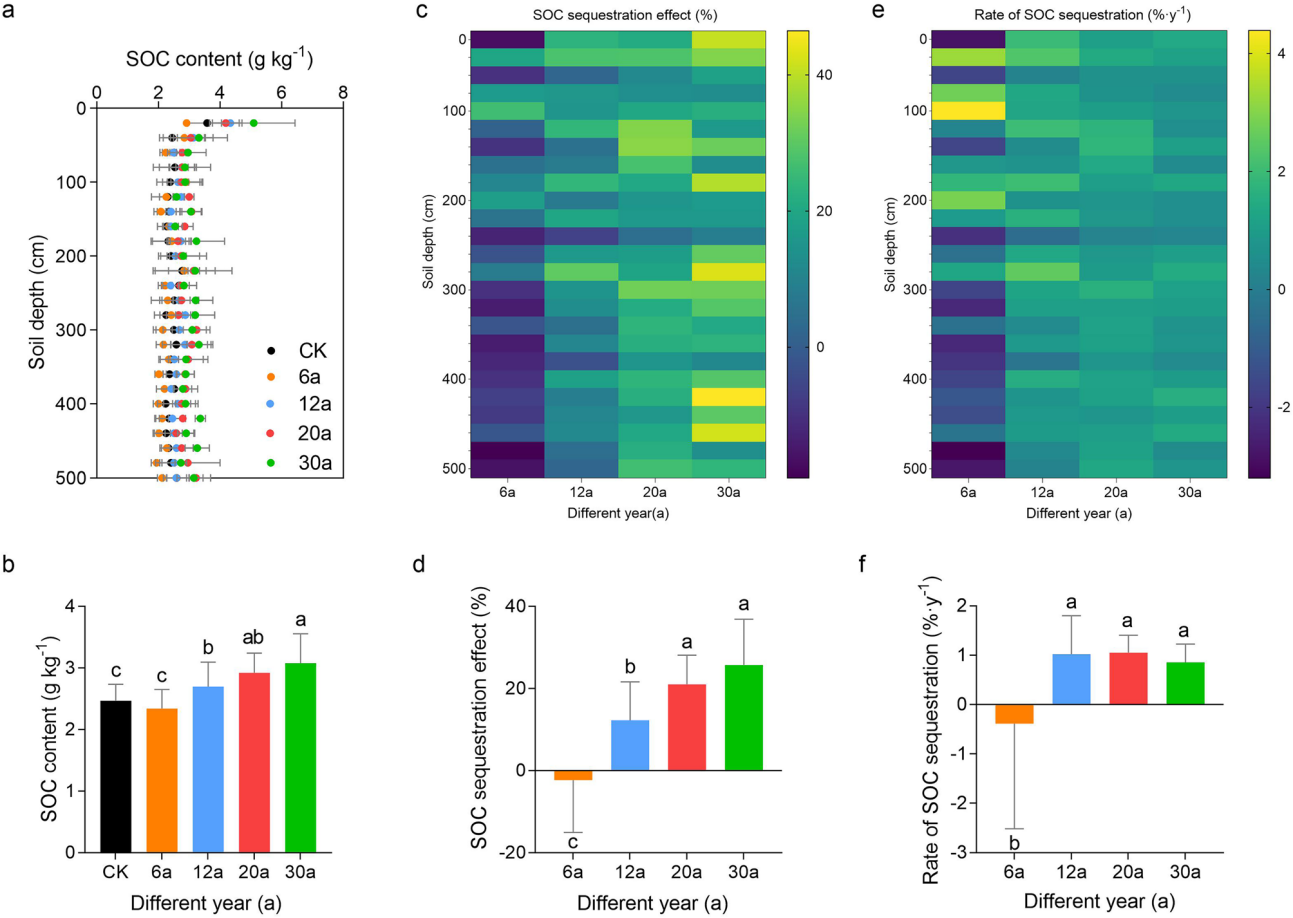
Vertical distribution of the SOC content (**a**,**b**) and SOC sequestration effect (**c**,**d**) and the rates of the SOC sequestration (**e**,**f**) in AOs of different stand ages. Error bars represent the standard error of the mean (n = 3).

Age and soil depth had an impact on the SOC content in the AOs, and the overall SOC content increased with age (Fig. 4a,b; Table 1). Although the SOC content of AOs-6 decreased (*p* > 0.05; Fig. 4b), it was no statistical difference from that of AL. Both AL and AOs of different ages exhibited “surface aggregation” as indicated by their SOC content. That is, the SOC content was greater within a range of 0–20 cm, while the SOC content at the other depths was less variable and showed some variation.

The SOCSE values in AOs varied with age and soil depth (Fig. 4c), with mean SOCSE values across the 0 to 500 cm profiles ranked as follows: AOs-30 (25.71%) > AOs-20 (21.02%) > AOs-12 (12.27%) > AOs-6 (− 2.31%). Soil carbon loss was observed in AOs-6 than in AL. The positive SOCSE in AOs occurred throughout the 0–500 cm layer and varied in different depths.

In terms of the carbon sequestration rate, the AOs of different ages were ranked as AOs-20 (1.05%) > AOs-12 (1.02%) > AOs-30 (0.86%) > AOs-6 (− 0.38%) (Fig. 4e,f). The carbon sequestration rates of AO-12 and AO-20 were the fastest and most concentrated, respectively (*p* < 0.05; Fig. 4f).

### Relationships between SMC, SOC, SMDE, SOCSE and RDWD

**Figure 5:**
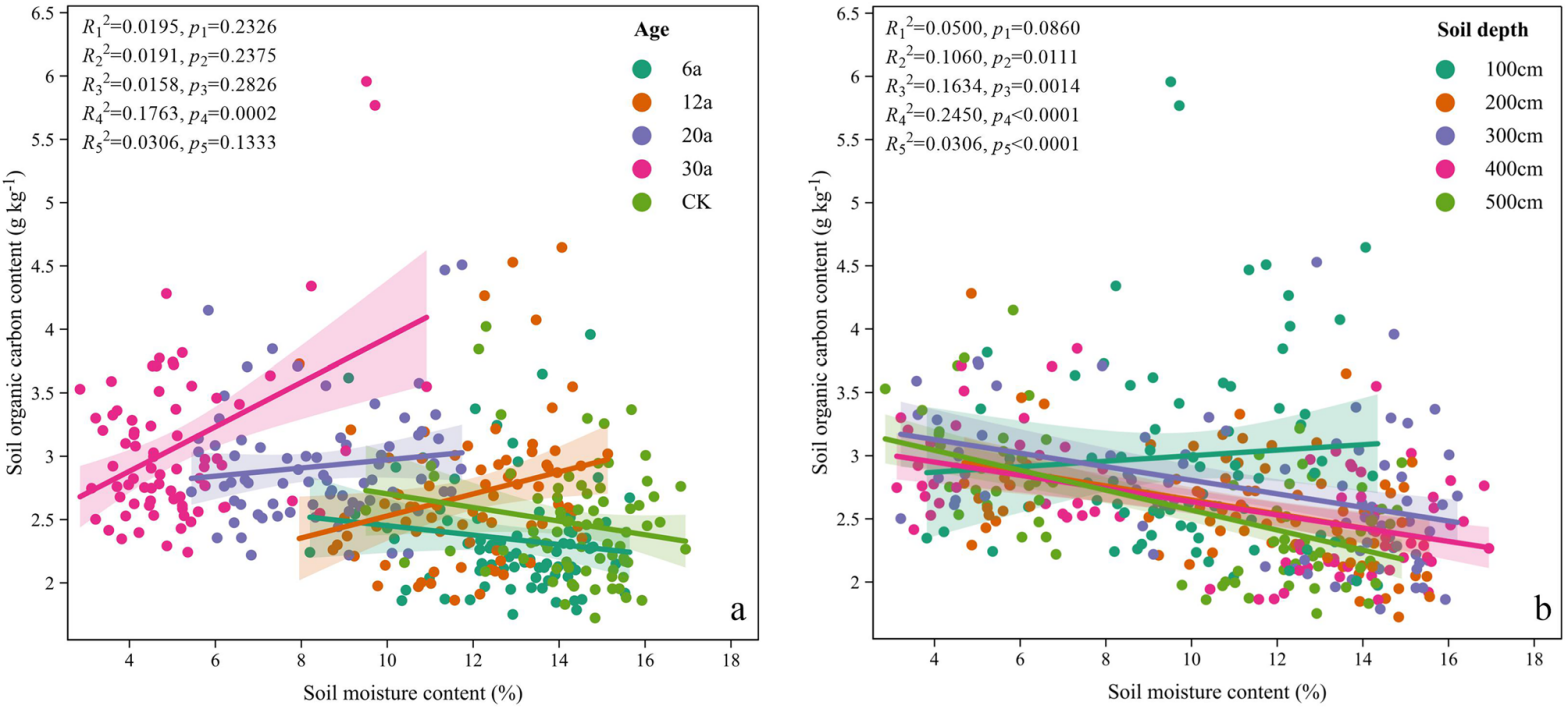
The relationship between SMC and SOC content in AOs of different stand ages and soil depths.

The relationships between SMC and SOC in AOs varied with age and soil depth (Fig. 5). Although we detected a positive and significant correlation between SMC and SOC at AOs-30 (*p* < 0.01; Fig. 5), we did not find any relationship between the two at AOs-6, AOs-12, AOs-20 or in the AL (*p* > 0.05). In regard to soil depth, we found no correlation between SMC and SOC at the 0–100 cm depth (*p* > 0.05; Fig. 5) but did find a significant negative correlation at 100–200, 200–300, 300–400 and 400–500 cm (*p* < 0.01), and this negative correlation became more significant with increasing soil depth (*p* < 0.01; Fig. 5).

**Figure 6:**
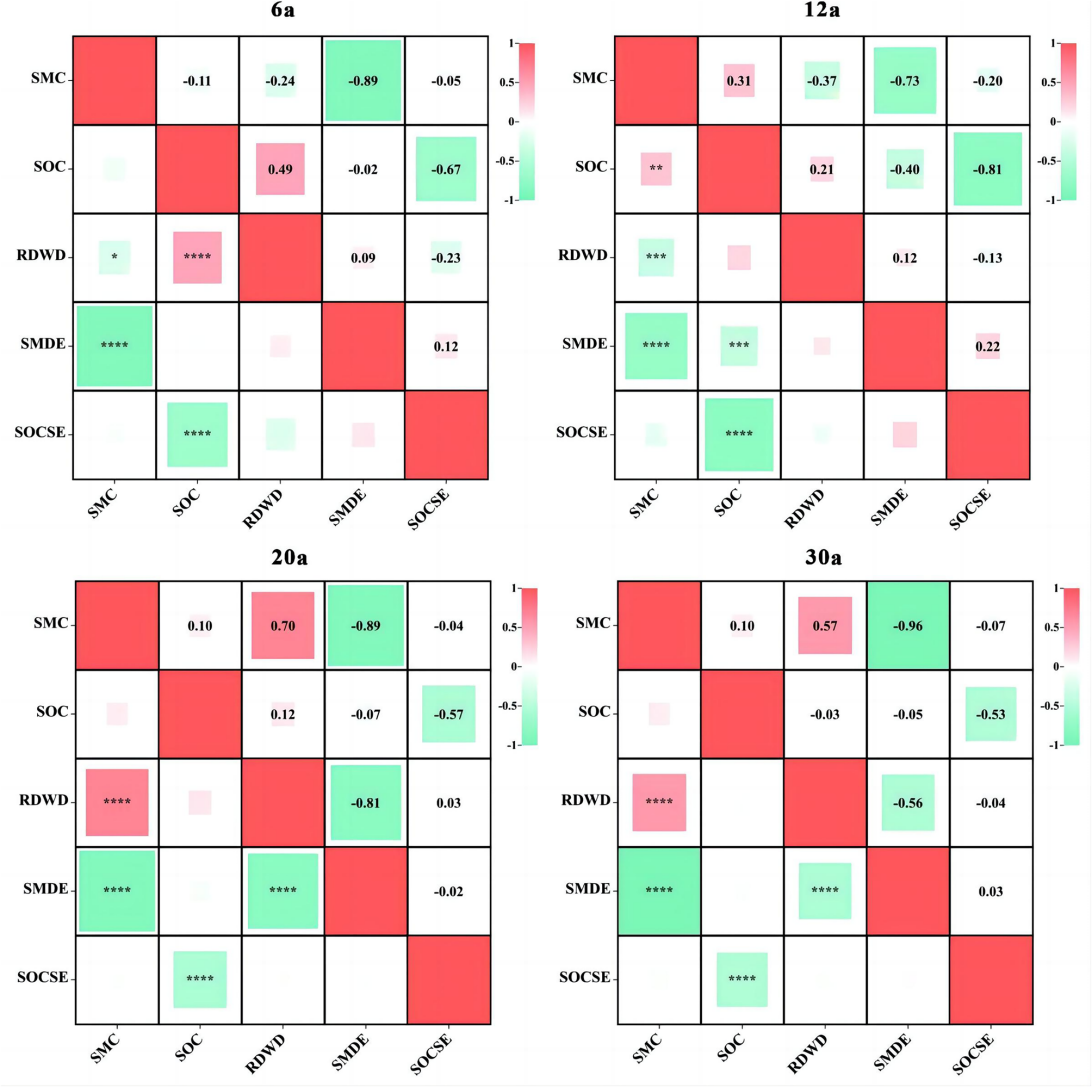
The relationship between SMC, SOC, SMDE, SOCSE and RDWD in the apple orchard varies with stand age. (*p < 0.05; **p < 0.01; ***p < 0.001; ****p < 0.0001). The SMC, SOC, SMDE, SOCSE and RDWD represent soil moisture content, soil organic carbon, soil moisture deficit effect, soil organic carbon sequestration effect and root dry weight density, respectively.

**Figure 7:**
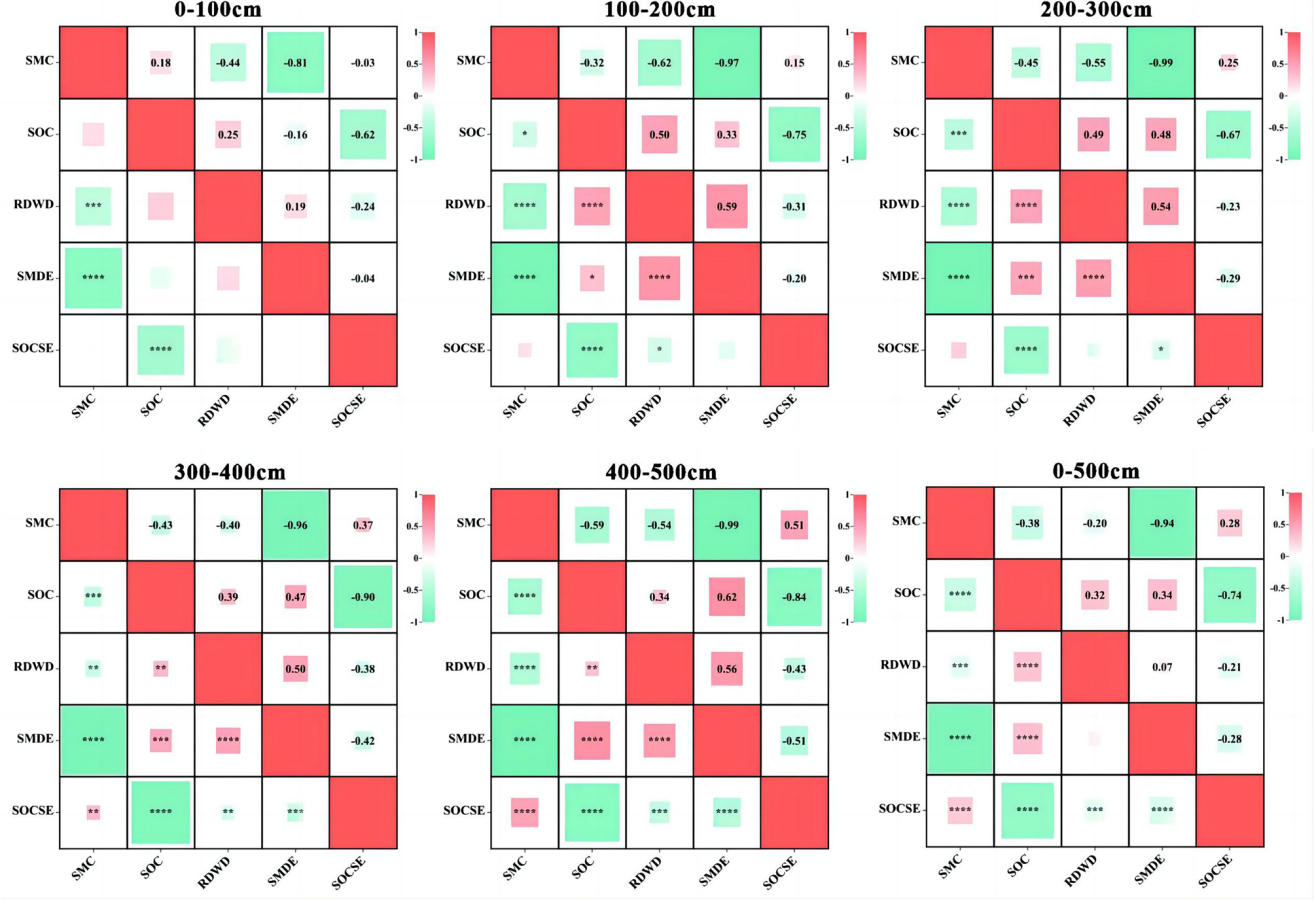
The relationship between SMC, SOC, SMDE, SOCSE and RDWD in the apple orchard varies with soil depth. (*p < 0.05; **p < 0.01; ***p < 0.001; ****p < 0.0001). The SMC, SOC, SMDE, SOCSE and RDWD represent soil moisture content, soil organic carbon, soil moisture deficit effect, soil organic carbon sequestration effect and root dry weight density, respectively.

The relationships between SMC, SOC, SMDE, SOCSE and RDWD in the AOs differed with age and soil depth (Figs. 6 and 7). In terms of age, the SMCs in AOs-6 and AOs-12 were negatively correlated with RDWD, and the SMCs in AOs-20 and AOs-30 were positively correlated with RDWD (*p* < 0.01; Fig. 6). SOC and RDWD showed no correlation (*p* > 0.05; Fig. 6). However, SMC was negatively correlated with RDWD at different soil depths (*p* < 0.05). We detected a positive correlation between 100–200, 200–300, 300–400 and 400–500 cm (*p* < 0.01) except at the 0 ~ 100 cm depth. In addition, we found a positive correlation between the SMDE and SOC and RDWD in all layers (100–500 cm) with the exception of the 0 ~ 100 cm depth (*p* < 0.01; Fig. 7).

## Discussion

### The impact of apple orchards on SMC

Vegetation affects soil moisture mainly through root uptake, utilization and canopy retention^28,29^. Different methods of vegetation restoration and years of restoration significantly affect hydrological processes. The SMC (0–500 cm) of the AOs in our research was significantly lower than that of AL, and varied with age and soil depth (Table 1), and decreased with increasing age (Fig. 3a,b), which is in agreement with previous findings^9,30,31^. Most likely, AL has lower evapotranspiration, while AOs, with a deep-rooted tree species, require more water uptake to maintain their growth, and this demand increases with the age of the stand (Fig. 2). Lower soil moisture drives apple tree roots to reach greater depths and extract deeper moisture to support their growth^30,32,25^.

We further observed that soil moisture deficits occurred in different layers in AOs of different ages, with shallower layers in younger AOs and deeper layers in older AOs (Fig. 3c,d), which is consistent with previous findings^33^. The main reason for the difference is that soil moisture depletion is mainly affected by vegetation transpiration and soil evaporation in apple orchards. During the early growth stage, evapotranspiration is low and precipitation can replenish the surface layer of the moisture loss, and soil moisture reserves of shallow layer can meet plant growth needs^34^. In the later stage, with the growth of apple trees, the shallow soil layer cannot meet its growth demand during the fruiting period especially. To maintain more aboveground biomass, the root system will inevitably penetrate deeper into the soil layer to absorb more deep soil moisture^35^. For instance, Bao et al.^36^ demonstrated that 5-year-old AOs did not drastically deplete SMC, 10-year-old AOs moved deep soil moisture to compensate for moisture deficits due to their own growth and insufficient rainfall, and 17-year-old AOs were under drought stress.

### The impact of apple orchards on SOC

The dynamics of SOC in our research differed with age and soil layer (Table 1, Fig. 4). Aboveground vegetation can influence the distribution and dynamics of SOC through a variety of complex ecological pathways^35,37^. The SOC content of both AL and AOs of different ages showed a clear "surface aggregation", which suggested that the growth of the AOs contributed to carbon sequestration in the top layer of the soil. This is mainly because surface litter is an essential source of surface SOC, and the seasonal shedding of apple branches and leaves provides a large amount of litter to the surface soil. This finding has been confirmed by many studies^31,25,38^. Moreover, a large amount of SOC was sequestered in deeper soil layers (> 100 cm) (Fig. 4), similar to previous research^25,38–40^. This indicates that deep soils have great potential for carbon sequestration^41,42^.

SOC content first decreased at the beginning of AO growth (AOs-6) (*p* > 0.05; Fig. 4b), while SOC showed an increasing trend at 12 a, 20 a and 30 a as the AOs grew, probably as a consequence of increased accumulation of organic matter inputs to the soil from aboveground litter as well as from belowground roots^43–45^. AOs-6 had the lowest SOC, probably because the strong soil disturbance caused by the planting of AOs stimulated a relatively stable soil environment before planting, increasing the release of SOC, and the lower initial aboveground and belowground biomass could not compensate for this difference^46,47^. Roots input more carbon to deeper soil, which may be the reason for the greater SOC sequestration in AOs-12 and AOs-20^46,48^. Since AOs-12 and AOs-20 are in the period of vigorous growth on the Loess Plateau, the root system must reach deeper soil layers to draw water, and root reach and growth are significant sources of organic carbon to deeper soils because root litter and its exudates are the main sources of organic carbon in deeper soils^42,47^. In addition, we detected no correlation between the SMDE and RDWD at 0–100 cm, whereas we detected significant positive correlations between the two in the 100–200, 200–300, 300–400, and 400–500 cm soil layers (*p* < 0.05), which could also indicate that the increase in root biomass consumes a large amount of SMC (Fig. 7). Therefore, we hypothesized that deep SOC sequestration in AOs comes at the cost of soil moisture depletion. In addition, although there was an increase in the SOC of AOs-30, it did not significantly differ from that of AOs-20 (*p* > 0.05; Fig. 4b). It is possible that the formation of a severe soil moisture deficit layer under older AOs inhibits root growth and slows the rate of fine-root turnover^45,46^. Dry soils may also promote the formation of cork lipids and lignin, which inhibits the release of root exudates and reduces the amount of organic matter available to deeper soils, limiting the sequestration of organic carbon in deeper soils^47,48^.

### Relationships between soil moisture, soil organic carbon and root parameters

It is well known that in the Loess Plateau with insufficient rainfall, the effective use of soil moisture is crucial for SOC sequestration^49^, whereas time and depth are essential factors affecting changes in soil water deficit and SOC sequestration (Table 1). In this research, there was no correlation between SMC and SOC in the 0–500 cm layer in the remaining AOs and AL, except in AOs-30, where SMC was positively correlated with SOC (Fig. 4). This is closely related to the 'time-dependent' nature of SOC sequestration under revegetation^50,51^. The relationship between SMC and SOC can only be demonstrated when the soil moisture deficit reaches a threshold that affects SOC sequestration, particularly where water is scarce. This finding has been confirmed by previous research findings^47,52,53^. The sources of soil organic carbon mainly include above and belowground plant residues and soil microbial residues; shallow soil organic carbon mainly comes from aboveground litter, and root secretions and residue are the most important sources of deep soil organic carbon^44,45^. Soil moisture affects soil organic carbon mainly through changes in aboveground plant photosynthesis and belowground root biomass and secretions^47,48^. In addition, soil moisture also affects soil organic carbon indirectly by influencing soil microbial biomass and extracellular enzyme activities. However, plants respond differently to different levels of soil moisture deficit. For example, Poorter et al.^52^ concluded that under moderate drought conditions, plant growth patterns changed little, root biomass increased only slightly relative to total biomass [root mass fraction (RMF)], and plants appeared to maintain their aboveground growth for as long as possible, thereby maintaining their competitiveness for aboveground resources. In contrast, plants exposed to severe drought experience a dramatic decrease in biomass and a large increase in RMF. Gaul et al.^53^ demonstrated that mild drought stimulated fine root production, whereas no renewal of fine root death was observed under drier conditions. Cai et al.^54^ found that fine root biomass and production under plantation forests were positively correlated with stand age, and that as soil moisture continued to decrease, the growth and renewal of fine roots were stimulated. All these studies illustrated that the degree of soil moisture deficit strongly influenced the characteristics of deep soil organic carbon sources, indirectly altering the relationship between SMC and SOC^48,54^. The behaviour of this alteration may be completely different between different climatic zones and forest types^55,26^.

At different soil depths, we did not detect a correlation between SMC and SOC in the 0–100 cm soil layer (*p* > 0.05; Fig. 5), but in the 100–200, 200–300, 300–400 and 400–500 cm soil layers, we detected a significant negative correlation between SMC and SOC (*p* < 0.05), and the negative correlation became more significant with increasing soil depth (*p* < 0.01; Fig. 5). This indicates that soil organic carbon sequestration in AOs is more sensitive to deep soil moisture conditions^31,25^. The negative correlation between the SMDE and SOCSE also became stronger with increasing soil depth (Fig. 7), suggesting that SOC sequestration in deeper layers of apple orchards is limited by soil moisture deficit. This finding is consistent with the results of previous related studies^31,25,49^. In addition, a positive correlation between SMC and SOC was also confirmed, contrary to our findings. For example, Feng et al.^49^ reported that soil moisture was significantly positively correlated with SOC at greater soil depths. Yang et al.^31^ reported that SMC in AOs was negatively correlated with SOC at depths of 0–100, 100–200 and 400–500 cm on the Loess Plateau, while the two were positively correlated at depths of 200–300 and 300–400 cm^27^. Li et al.^14^ showed that the SMC and SOC were positively correlated in young AOs, whereas no correlation was found between them in old AOs, and the completely different coupled relationships between SMC and SOC may be caused by differences in climatic conditions, vegetation types and growth stages.

**Figure 8:**
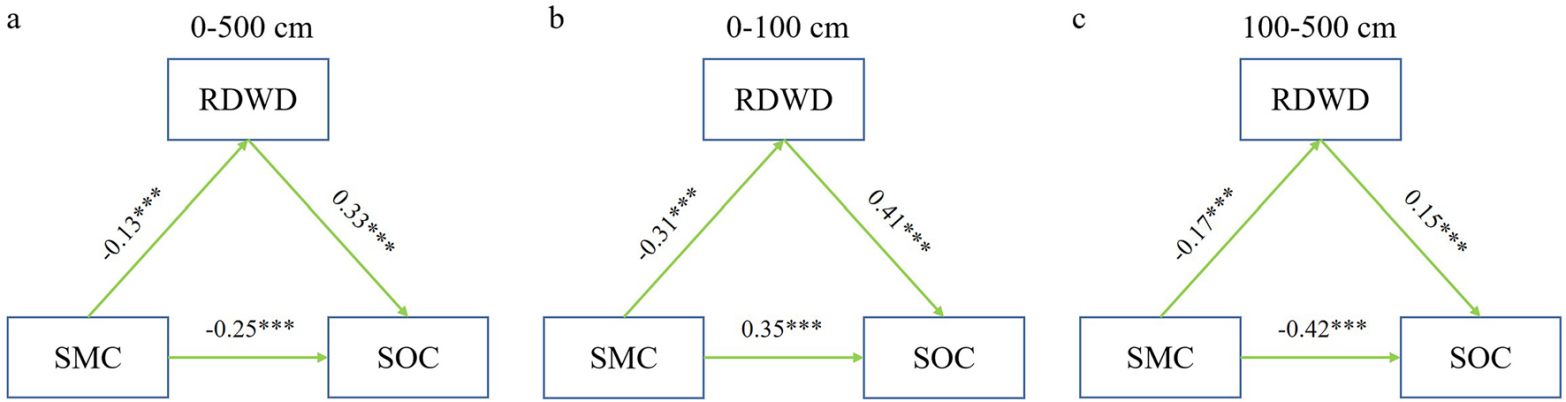
Path analysis between SMC, RDWD and SOC at different soil depth. The green arrow and the numbers above it indicate the normalized direct effect and the normalized path coefficients, respectively (*p < 0.05; **p < 0.01; ***p < 0.001).

In our research, SMC was negatively correlated with RDWD in AOs-6 and AOs-12, and SMC was significantly positively correlated with RDWD in AOs-20 and AOs-30 (*p* < 0.01; Fig. 6). This may be related to different soil moisture utilization strategies in AOs at different stages^26,32^. The increase in rooting depth and biomass of apple trees in the early stage depleted SMC^33^, but the soil moisture deficit shifted this relationship with further growth of AOs. In the 0–500 cm soil profile, the SMC was negatively correlated with RDWD, which was closely related to the growth process of AOs, where the shortage of soil moisture promoted the increase of root depth and biomass, and the growth of the root system exacerbated the depletion of soil moisture^42,47^. Except for SOC and RDWD in AOs-6, which showed a positive correlation, SOC and RDWD in AOs of other ages showed no correlation (p > 0.05; Fig. 6). This indicates that the root system positively influences SOC sequestration in the early stage, and greater SOC also favours root growth. Then, with the increase of soil moisture deficit, it changed the relationship between the two^47,48^. There was a significant positive correlation between SOC and RDWD at the 100–200, 200–300, 300–400 and 400–500 cm depths (*p* < 0.01; Fig. 6), except at the 0 ~ 100 cm depth, where there was no correlation between SOC and RDWD. This suggests an important contribution of the root system to deep SOC accumulation^46,26^. The results of the throughput analyses in this study also showed that SMC directly affects SOC at different soil depths and regulates SOC by affecting RDWD, but there were significant differences in this effect at different depths (Fig. 8). In addition, considering the effects of deep soil moisture deficit on SOC sequestration and the important role of roots in the coupled relationship between the two factors, in-depth study of the physical, chemical and biological pathways through which deep soil moisture deficit affects SOC sequestration is a priority for future research.

### Implications for future dryland apple orchard management practices

**Figure 9:**
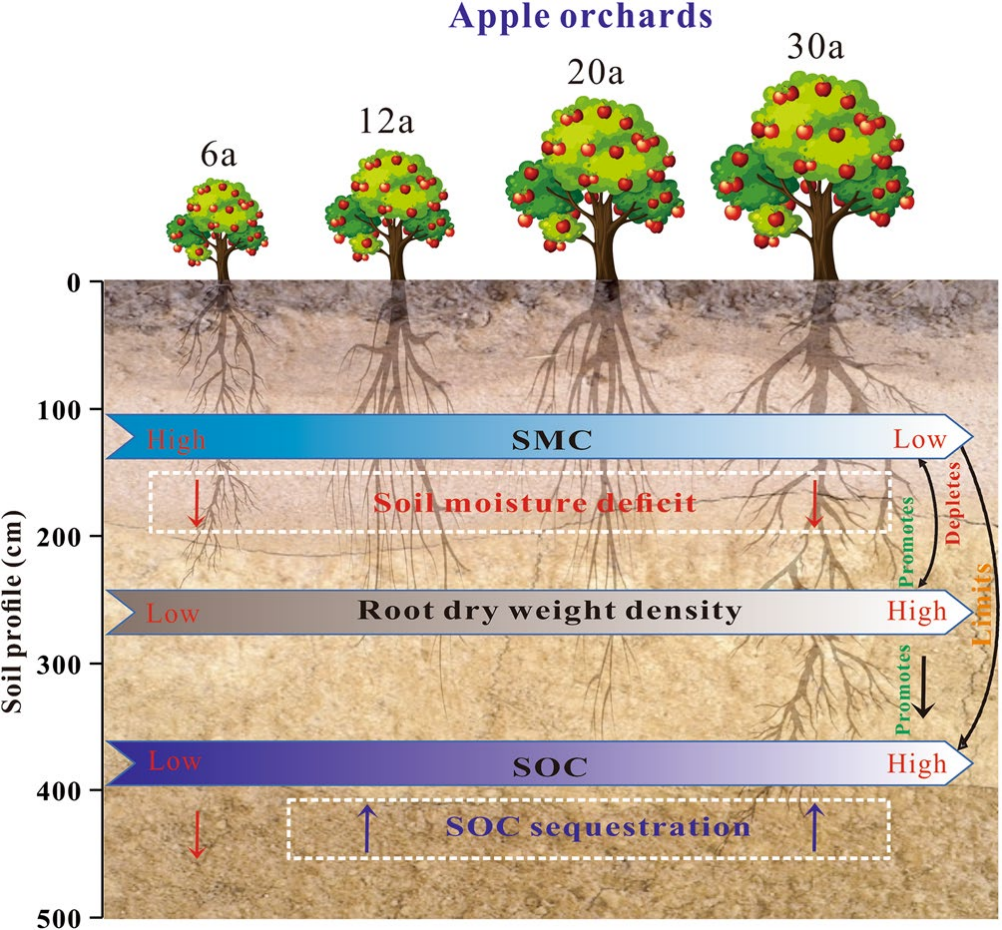
Conceptual diagram illustrating the effects of AOs (deep-rooted plants) on RDWD, SMC and SOC. The Wide arrows show the changes of RDWD, SMC and SOC with increasing of orchard age. The white rectangular dotted outline represents the age stage in the apple orchard where the phenomenon occurs. Up (in purple) and down (in red) arrows indicate increasing and decreasing trends, respectively. The black bidirectional arrows represent the interaction of two factors, and the black unidirectional arrows represent the effect of one factor on another factor.

Planting of deep-rooted tree species in the Loess Plateau region can lead to varying degrees of soil moisture deficit, decreased SOC sequestration and poor tree growth (Fig. 9). Although apples are a cash crop, deep-rooted AOs can lead to excessive use of deep soil water, which can affect the healthy and sustainable growth of AOs (e.g. apple quality, yield and soil carbon sequestration). In the water-scarce Loess Plateau region, the economic and ecological benefits of AOs must be properly weighed^56^. However, we can work to make this trade-off healthier, more efficient and synergistic. Root-induced carbon sequestration is often accompanied by significant soil moisture depletion. Therefore, we suggest that planting and design should carefully consider scientifically sound planting patterns and management practices that increase apple quality and yield, as well as soil carbon sequestration, at the expense of minimal SMC^51^. For example, intermediate cuttings, intercropping with herbaceous plants, and understory mulching promote soil moisture utilization efficiency and optimize the relationship between soil moisture supply and plant water requirements^57^. To reduce deep soil water depletion and ensure sustainable utilization of water, improving the efficiency of rainwater harvesting has also been proposed.

## Conclusions

We investigated the SMC deficit and SOC sequestration effects and the coupled between the SMC and SOC at different ages in AOs and characterized the role of roots in these changes. We observed that the SMC in the 0–500 cm profile of AOs tended to generally decrease with age, with varying degrees of SMD in all layers, in comparison with that of AL. The highest rate of SMD was found in AOs-20. Surface aggregation characteristics occurred in each AO, and the increase in SOC content with age. Deeper soils are also important for carbon sequestration, but carbon sequestration does not always occur, and carbon loss is also observed. The SMC deficit strongly affects SOC sequestration, and this effect is even more notable at deeper soil layers. Both Pearson correlation and path analysis indicated that SMC, SOC, and RDWD interact with each other and are constantly changing with time and soil depth. Our study emphasized the interactions among SMC, SOC and root system in deep layers of AOs. These findings may provide a basis for the management of healthy and sustainable practices in AOs in the Loess Plateau region.

## Data Availability

The datasets analyzed during the current study available from the corresponding author on reasonable request.
